# Supplementary material for: Dielectric ordering of water molecules arranged in a dipolar lattice
Source: Nat Commun. 2020 Aug 6;11:3927. doi: 10.1038/s41467-020-17832-y (PMC7411056; doi:10.1038/s41467-020-17832-y)
Supplement: Supplementary file 1 — Supplementary Information [file 41467_2020_17832_MOESM1_ESM.pdf]

Supplementary Information

Dielectric ordering of water molecules arranged in a dipolar lattice

Belyanchikov *et al.*

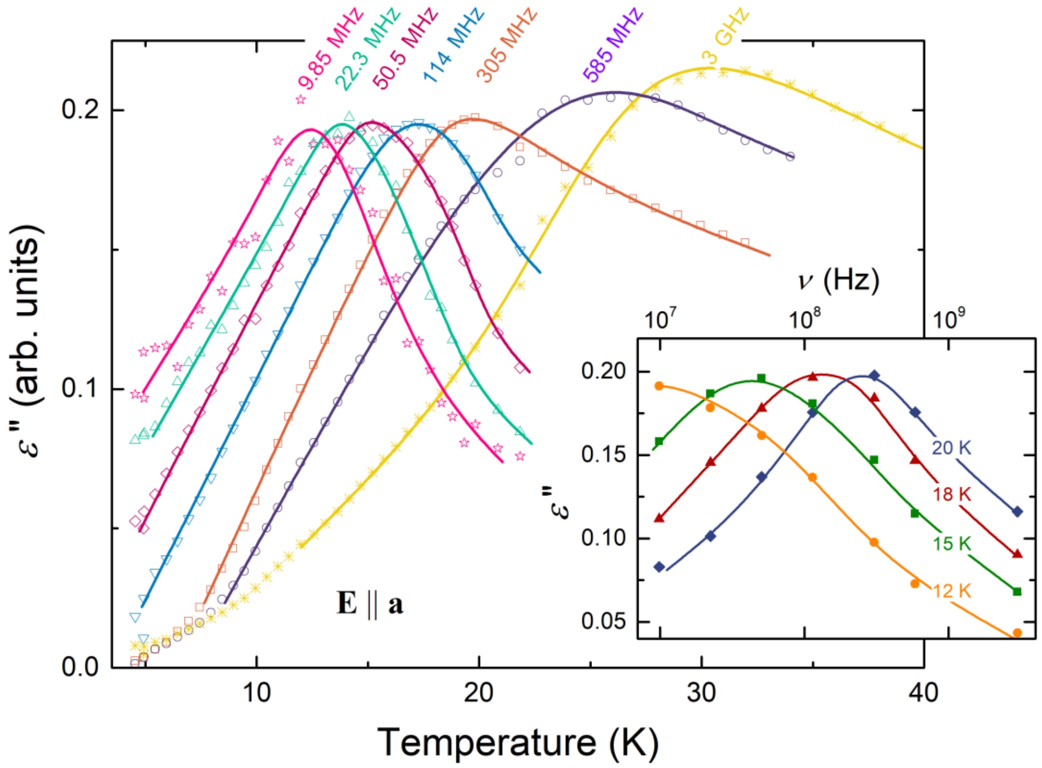

Supplementary Figure 1 | Radio-frequency and microwave dielectric losses. Temperature-dependent dielectric loss of water molecules confined in cordierite for **E** || **a** polarization, measured at various frequencies. Inset: loss spectra derived from these data at four temperatures as indicated. All lines are guides to the eye. While no absolute values are provided by the experiments, temperature-dependent relaxation times can be reliably deduced by the condition $\omega\tau = 1$, valid at the peaks in $\varepsilon''(T)$ and $\varepsilon''(\nu)$.

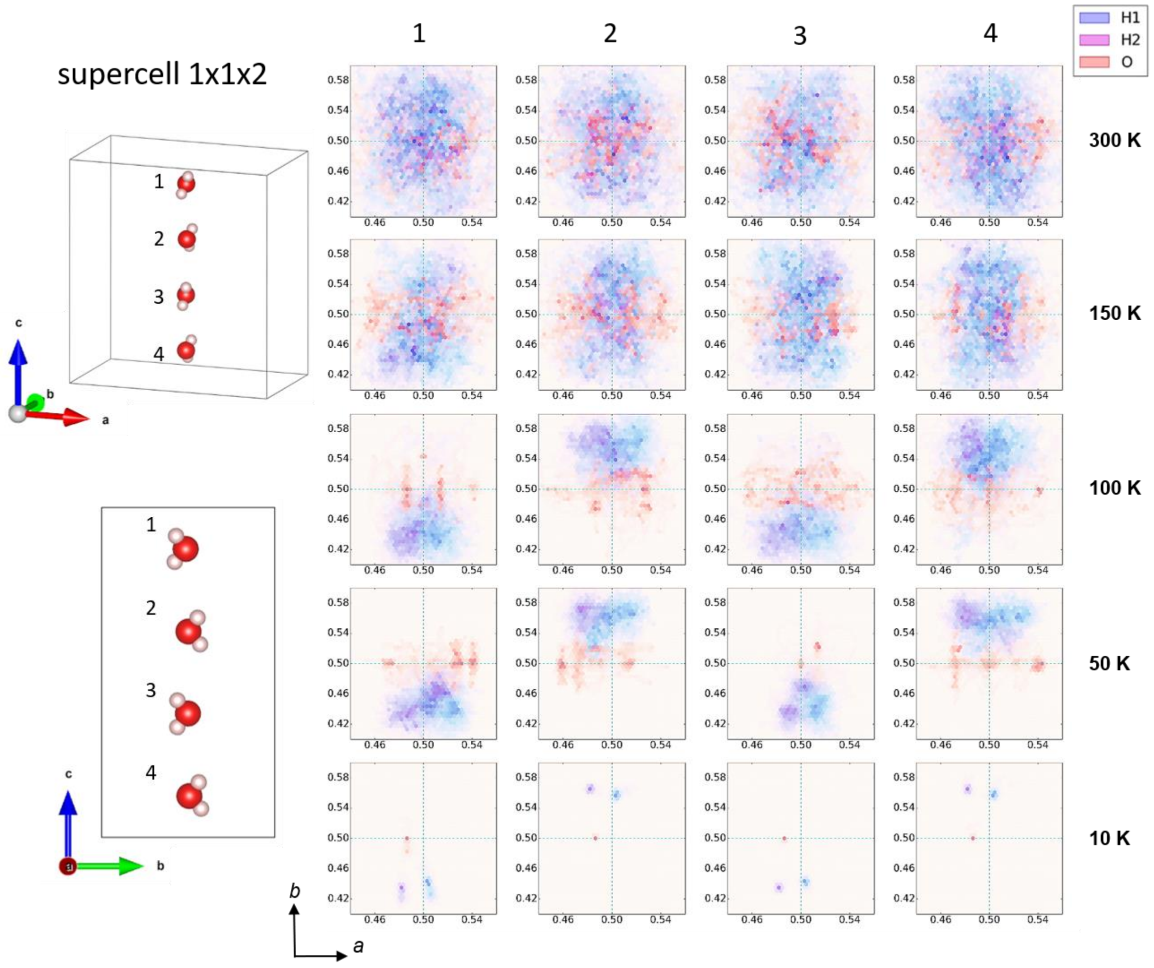

Supplementary Figure 2 | Temperature-dependent dynamics of nanoconfined water molecules in channel in 1x1x2 supercell. Density functional theory molecular dynamics simulation during 20 ps and at five temperatures of four water molecules within four nanocages of cordierite 1x1x2 supercell, located next to each other along the channel **c**-axis. The panels display projections of oxygen ions (O) and two protons (H1, H2) of the H_2O molecules on the **ab**-plane; the **c**-axis is perpendicular to the figure plane. Each column corresponds to one of four water molecules that show antiferroelectric alignment along the channel. Numbers correspond to the coordinates of atoms within the cages in fractions of translation vectors of cordierite unit cell.

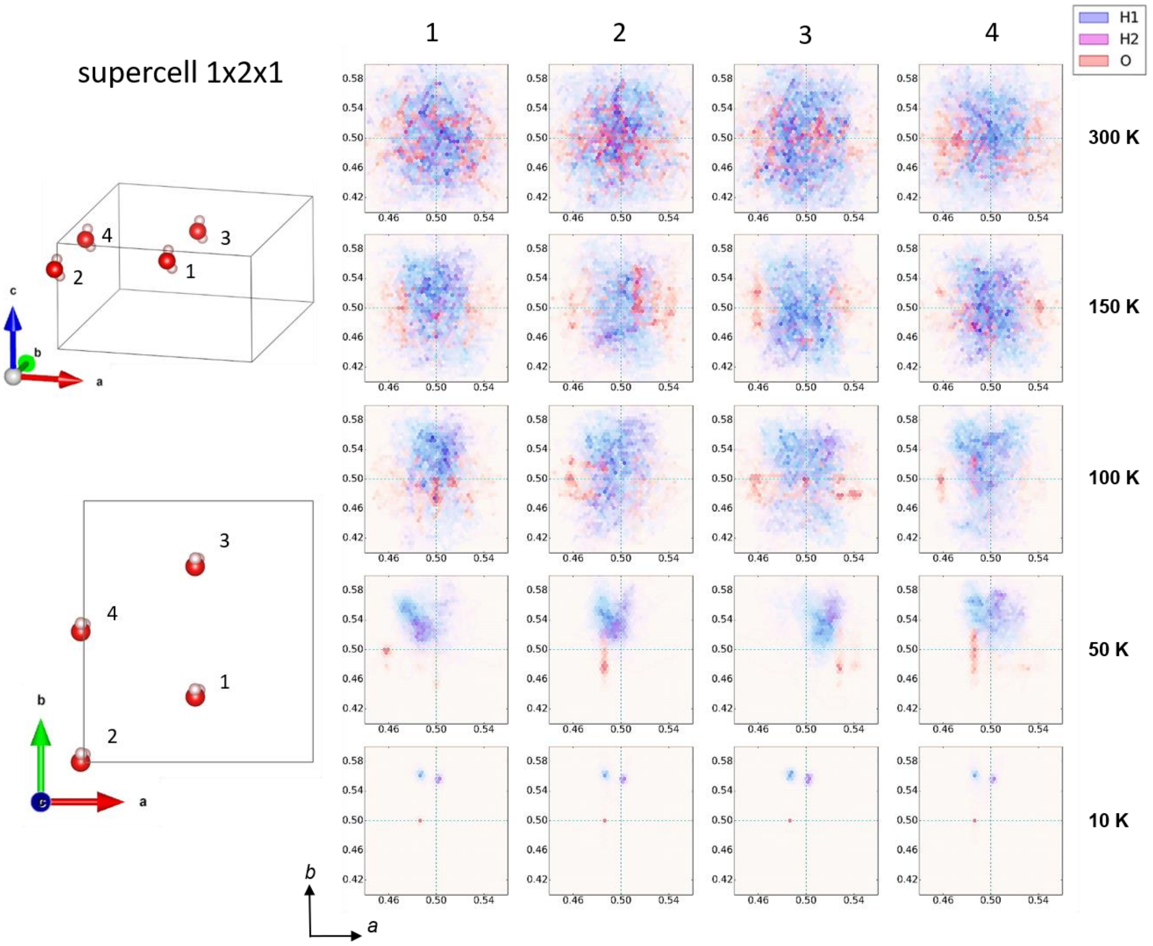

Supplementary Figure 3 | Temperature-dependent dynamics of nanoconfined water molecules in ab-plane in 1x2x1 supercell. Density functional theory molecular dynamics simulation during 20 ps and at five temperatures of four water molecules within four nanocages of cordierite 1x2x1 supercell, located next to each other within the **ab**-plane. The panels display projections of oxygen ions (O) and two protons (H1, H2) of the H_2O molecules on the **ab**-plane; the **c**-axis is perpendicular to the figure plane. Each column corresponds to one of four water molecules that show ferroelectric alignment in the **ab**-plane. Numbers correspond to the coordinates of atoms within the cages in fractions of translation vectors of cordierite unit cell.

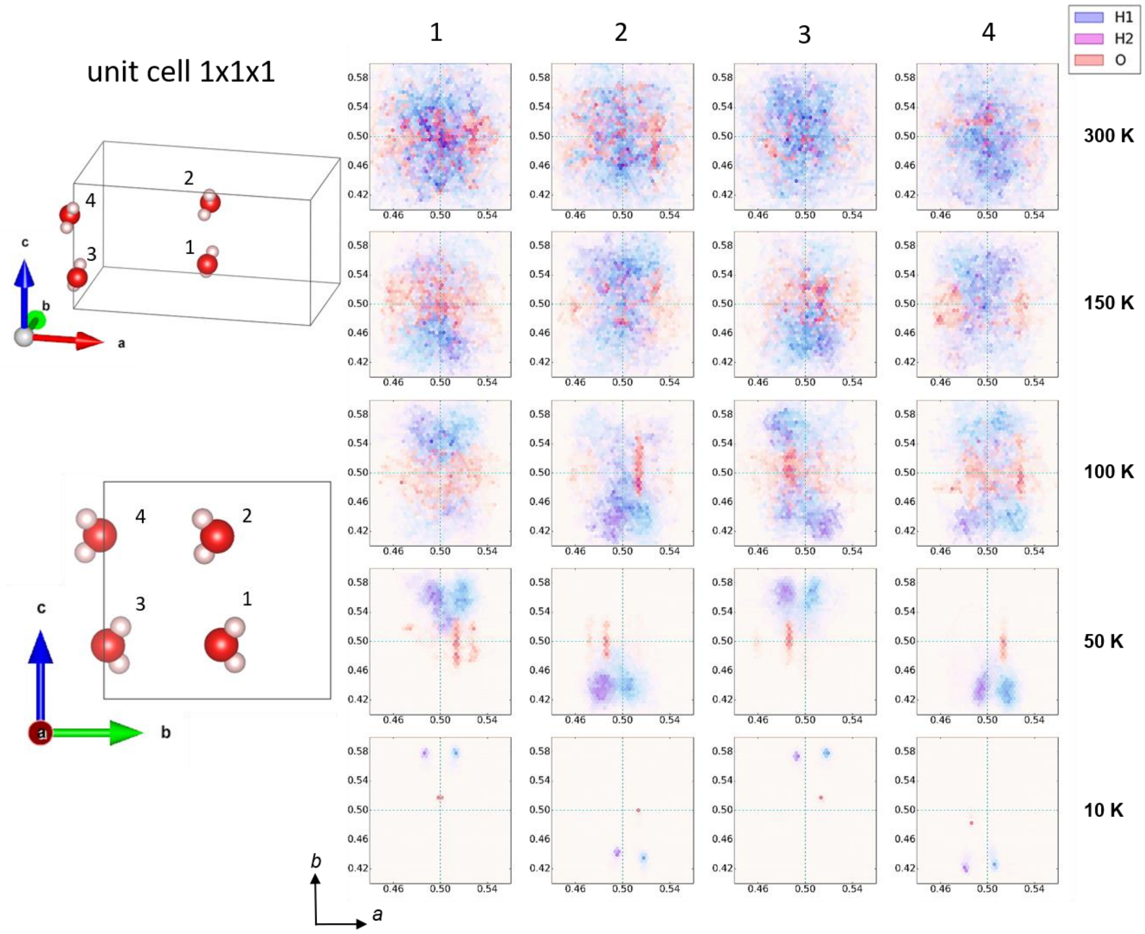

Supplementary Figure 4 | Temperature-dependent dynamics of nanoconfined water molecules in the unit cell of cordierite crystal. Density functional theory molecular dynamics simulation during 20 ps and at five temperatures of four water molecules within four nanocages of cordierite crystal unit cell. The panels display projections of oxygen ions (O) and two protons (H1, H2) of the H_2O molecules on the **ab**-plane; the **c**-axis is perpendicular to the figure plane. Each column corresponds to one of four water molecules that show antiferroelectric alignment along the channel (1, 2 and 3, 4 molecule) and ferroelectric alignment for molecules in the **ab**-plane (1, 3 and 2, 4 molecule). Numbers correspond to the coordinates of atoms within the cages in fractions of translation vectors of cordierite unit cell.

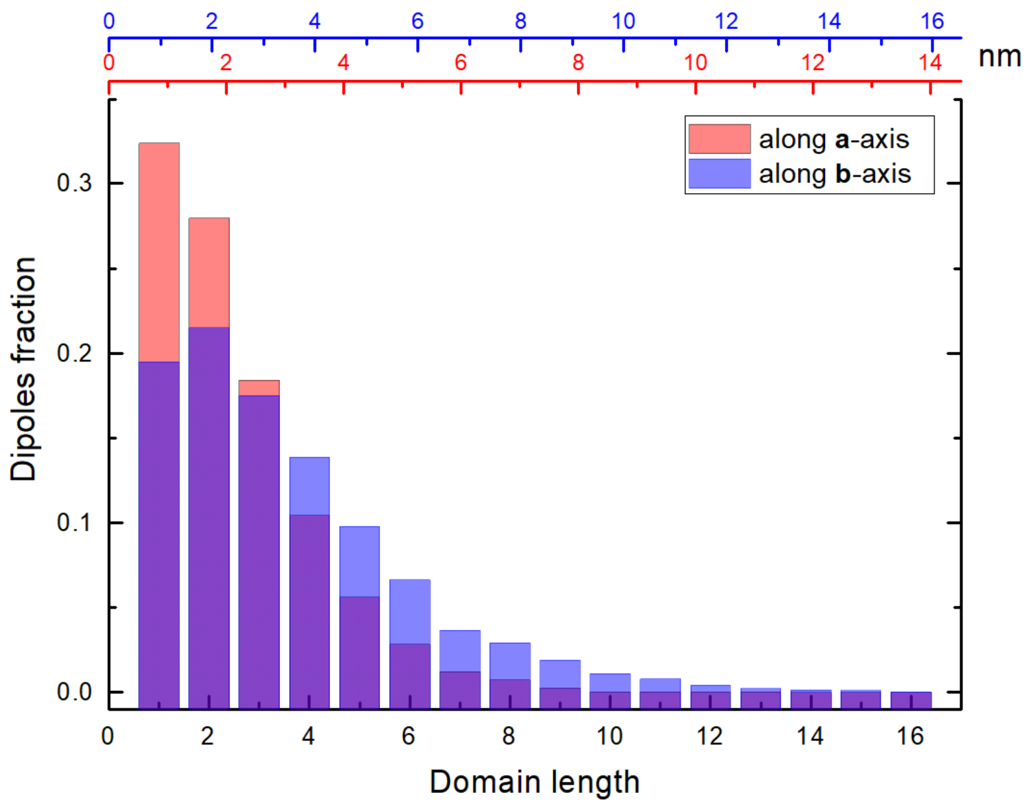

Supplementary Figure 5 | Distribution of ground state domains sizes along the a- and b-axes as a function of domains length from Monte Carlo simulation. Domains are composed by collinear dipoles in same **ab**-plane; their boundaries are given by dipoles of different directions, by defects or boundaries of the sample. Calculations are done for sample of dimensions 16x16x16 and 75% H_2O filling factor. The vertical axis corresponds to fraction of overall water dipoles in every domain size. The domains length is given in the units of dipole sites (bottom axis) and in nanometers along **a**-axis (upper red) and **b**-axis (upper blue).

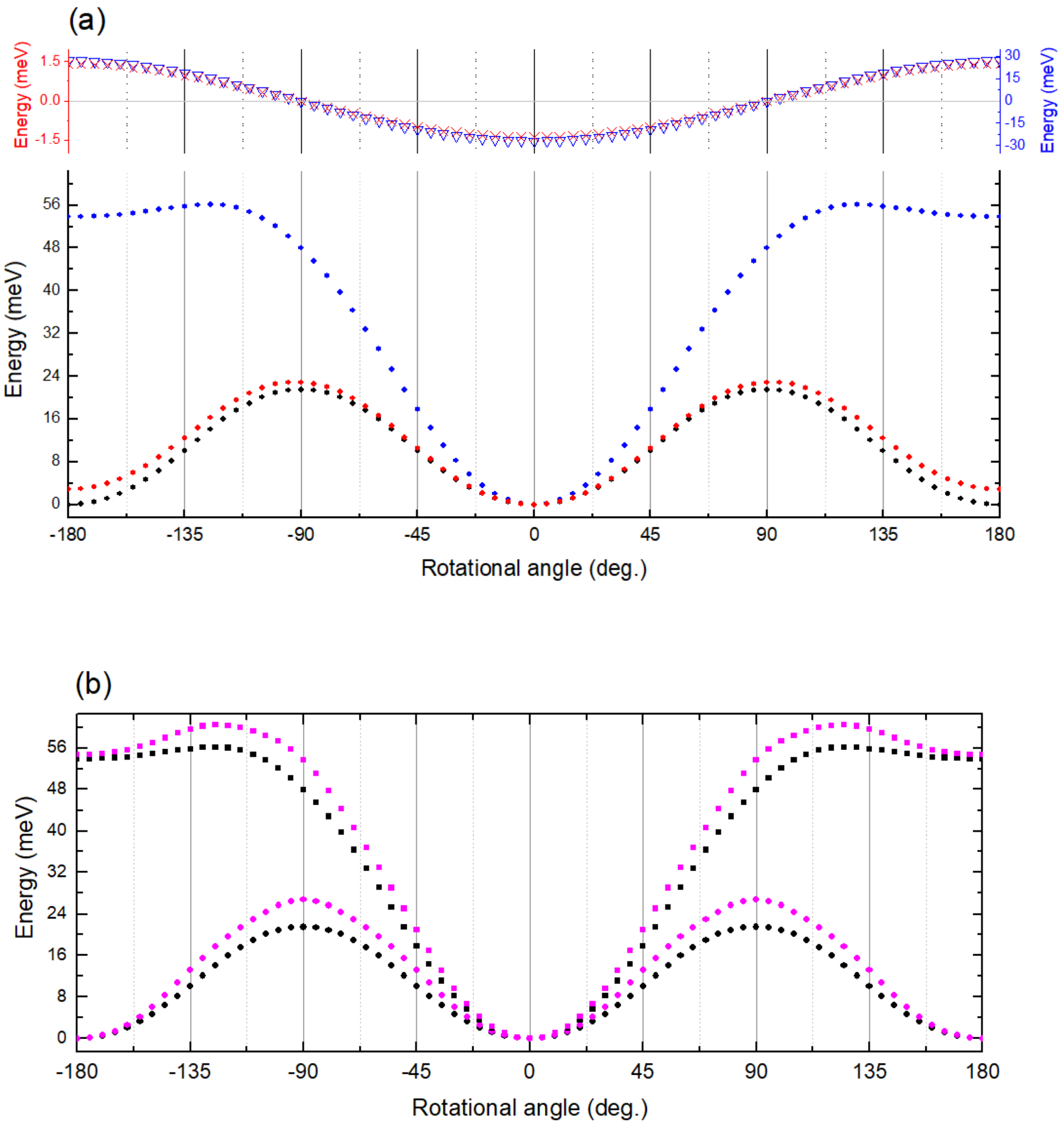

Supplementary Figure 6 | Potential energy relief for rotational degree of freedom of nanoconfined water molecule. (a) Rotational potential of single nanoconfined water molecule in cordierite (black dots), of the same water molecule additionally interacting with another water molecule placed in neighboring cage in the **ab**-plane (red dots) and in the channel (blue dots). Crosses and triangles show dipolar coupling between the two molecules in neighboring cages in plane and in channel, respectively, obtained by subtraction of crystalline potential. Note different Y-scales. (**b)** Perdew–Burke–Ernzerhof (black dots) and van der Waals-corrected (magenta dots) rotational potentials for single nanoconfined water molecule (circles) and for separate water molecule in completely filled unit cell containing four molecules (squares).

Supplementary Table 1 | X-ray single-crystal diffraction analysis of cordierite crystal at 85 K. Coordinates of cordierite atoms and water oxygen atom, position occupancy *Q*, equivalent thermal parameters U_{eq}.

Atom	Site	*x/a*	*y/b*	*z/c*	*Q*	U_{eq}
Mg	8*g*	0.337464(4)	0	1/4	0.925(1)	0.00390(2)
Fe	8*g*	0.337464(4)	0	1/4	0.065(1)	0.00390(2)
Al3	8*g*	0.337464(4)	0	1/4	0.007(1)	0.00390(2)
Al1	8*k*	1/4	1/4	0.25019(1)	0.967(2)	0.00334(2)
Si4	8*k*	1/4	1/4	0.25019(1)	0.033(2)	0.00334(2)
Al2	8*l*	0.050643(6)	0.307675(1)	0	1.0	0.00296(2)
Si1	4*b*	0	1/2	1/4	1.0	0.00296(2)
Si2	8*l*	0.192137(6)	0.078117(2)	0	1.0	0.00276(2)
Si3	8*l*	0.135094(6)	0.763310(2)	0	1.0	0.00277(2)
O1	16*m*	0.246716(10)	0.896769(6)	0.358474(2)	1.0	0.00469(4)
O2	16*m*	0.062038(10)	0.584173(6)	0.34889(2)	1.0	0.00451(4)
O3	16*m*	0.826794(1)	0.690658(6)	0.358228(2)	1.0	0.00475(4)
O4	8*l*	0.043197(1)	0.752702 (9)	0	1.0	0.00645(5)
O5	8*l*	0.121878 (1)	0.184331 (9)	0	1.0	0.00627(6)
O6	8*l*	0.164361 (1)	-0.079187 (9)	0	1.0	0.00619(6)
Na	4*c*	0	0	0	0.029(3)	0.0125(6)
K	4*c*	0	0	0	0.008(3)	0.0125(6)
C1	4*a*	0	0	1/4	0.106(1)	0.0034(6)
O8	16*m*	0.067277(6)	0.011790(6)	0.2559(6)	0.053(5)	0.0115(8)
O7	8*g*	0.011401(7)	0	1/4	0.092(6)	0.0771(6)
H1	16*m*	0.035663(9)	0.057671(6)	0.167357(9)	0.046(7)	0.0158(9)
H2	16*m*	0.035112(9)	0.052437(6)	0.327004(9)	0.046(7)	0.0158(9)
Ca	8*g*	0.3373(6)	0	1/4	0.001(1)	0.0039(12)
Mn	8*g*	0.3373(6)	0	1/4	0.001(1)	0.0039(12)

Supplementary Note 1 | Dipolar coupling.

To estimate dipolar couplings between water molecules within cordierite nanocages we approximated them by rigid rotors in a rigid lattice (i.e. one degree of freedom per molecule). More specifically, an undistorted H_2O molecule (gas-phase geometry) was placed at the center of nanocage of relaxed cordierite crystal in such a way that the H-H vector was parallel to the **c**-axis which in turn was crossing the middle point between oxygen and two protons. The rotational angle is defined in such a way that 0° angle of each molecule corresponds to the ground state geometry. Then the one-dimensional potential energy surface (PES) of a rotor is obtained by rigid DFT-energy scan in three settings: (a) separate water molecule rotated to study the crystal potential; (b) two neighboring molecules placed along the channel **c**-axis and (c) two neighboring molecules in the **ab**-plane, cases (b) and (c) used to study dipolar coupling between the molecules. For two molecules, the potential energy surface was determined by changing orientation and position of a separate molecule while the other molecule was kept fixed. The results are shown in Supplementary Fig. 6(a). Black dots show rotation potential for a separate molecule in the unit cell that characterizes interaction of the molecule with the ions that form nanocage. There are two minima with the energy barrier of 21.5 meV in-between. The barrier height is somewhat larger than that obtained from estimations of activation energy. The discrepancy is due to the fact that only one of 7 degrees of freedom of water molecule was considered and simple rotation of the molecule considered here is not the most energetically favorable path between two energy minima. Nevertheless, that way allows to easily estimate the strength of dipolar coupling between water molecules. Red and blue dots in Supplementary Fig. 6(a) show PES of a molecule rotating in presence of another fixed molecule in the neighboring cage within the **ab**-plane and in the channel, respectively. Subtraction of crystal potential (red crosses and blue triangles) provides with the potential that characterizes dipole coupling between two respective pairs of water molecules; both in plane and in channel rotation potentials perfectly follow sine function which corresponds to electrostatic dipolar coupling. Since the distance between the molecules in the **ab**-plane (≈ 10 Å) is larger than that in channels (≈ 5 Å), corresponding force constants 1.4 meV and 26.9 meV estimated from Supplementary Fig. 6(a) are also different.

To find out whether van der Waals (vdW) forces play any significant role in interaction between water molecules and ions of nanocage, we performed two test calculations using the non-local dispersion corrected vdWDF2 functional. Supplementary Fig. 6(b) shows rotation potentials for Perdew–Burke–Ernzerhof (PBE) functional (black dots) and for vdWDF2-corrected functional (magenta dots) for two cases: separate water molecule in the unit cell (circles) and completely filled unit cell containing four molecules (squares). It is seen that for both cases accounting for the vdW interaction does not change the potential shape but slightly changes its amplitude. It is important that after subtracting the crystal potentials for corresponding full filled cases, both full filled curves match with only negligible difference. Thus, including vdW correction does not affect the shape of the crystal potential and dipole coupling of water molecules, but changes the absolute value of the crystal potential experienced by water molecule.
