# Supplementary material for: Dielectric ordering of water molecules arranged in a dipolar lattice
Source: Nat Commun. 2020 Aug 6;11:3927. doi: 10.1038/s41467-020-17832-y (PMC7411056; doi:10.1038/s41467-020-17832-y)
Supplement: Supplementary file 3 — Description of Additional Supplementary Files [file 41467_2020_17832_MOESM3_ESM.pdf]

Description of Additional Supplementary Files

Supplementary Movie 1 | Molecular dynamics of nanoconfined water molecules in channel in the unit cell of cordierite crystal at 10 K. Density functional theory molecular dynamics simulation of two water molecules within two nanocages of cordierite crystal lattice, located next to each other along the channel **c**-axis. The video shows first 5 of 15 ps simulation performed with 1 fs time step and periodic boundary conditions.

Supplementary Movie 2 | Molecular dynamics of nanoconfined water molecules in channel in the unit cell of cordierite crystal at 100 K. Density functional theory molecular dynamics simulation of two water molecules within two nanocages of cordierite crystal lattice, located next to each other along the channel **c**-axis. The video shows first 5 of 15 ps simulation performed with 1 fs time step and periodic boundary conditions.

Supplementary Movie 3 | Molecular dynamics of nanoconfined water molecules in channel in the unit cell of cordierite crystal at 300 K. Density functional theory molecular dynamics simulation of two water molecules within two nanocages of cordierite crystal lattice, located next to each other along the channel **c**-axis. The video shows first 5 of 15 ps simulation performed with 1 fs time step and periodic boundary conditions.

Supplementary Movie 4 | Molecular dynamics of nanoconfined water molecules in ab-plane in the unit cell of cordierite crystal at 10 K. Density functional theory molecular dynamics simulation of two water molecules within two nanocages of cordierite crystal lattice, located next to each other within the **ab**-plane. The video shows first 5 of 15 ps simulation performed with 1 fs time step and periodic boundary conditions.

Supplementary Movie 5 | Molecular dynamics of nanoconfined water molecules in ab-plane in the unit cell of cordierite crystal at 100 K. Density functional theory molecular dynamics simulation of two water molecules within two nanocages of cordierite crystal lattice, located next to each other within the **ab**-plane. The video shows first 5 of 15 ps simulation performed with 1 fs time step and periodic boundary conditions.

Supplementary Movie 6 | Molecular dynamics of nanoconfined water molecules in ab-plane in the unit cell of cordierite crystal at 300 K. Density functional theory molecular dynamics simulation of two water molecules within two nanocages of cordierite crystal lattice, located next to each other within the **ab**-plane. The video shows first 5 of 15 ps simulation performed with 1 fs time step and periodic boundary conditions.
